# Proteasome inhibitor, ixazomib prevents topoisomerase‐I degradation and reverses irinotecan resistance in colorectal cancer

**DOI:** 10.1002/1878-0261.70256

**Published:** 2026-04-15

**Authors:** Yuho Ebata, Koji Ando, Hirofumi Hasuda, Koshi Mimori, Elizabeth C. Unan, Siddhartha Pulukuri, Aahana Tiku, Allison Berger, Eiji Oki, Ajit Bharti, Tomoharu Yoshizumi

**Affiliations:** ^1^ Department of Surgery and Science, Graduate School of Medical Sciences Kyushu University Fukuoka Japan; ^2^ Department of Gastroenterological Surgery and Clinical Research Institute Kyushu Medical Center Fukuoka Japan; ^3^ Department of Surgery Kyushu University Beppu Hospital Oita Japan; ^4^ Department of Medicine, Division of Hematology Oncology Boston University School of Medicine Boston MA USA; ^5^ Takeda Pharmaceutical Company Limited Cambridge MA USA; ^6^ Department of Advanced Medicine and Innovative Technology Kyushu University Hospital Fukuoka Japan; ^7^ Predictus Bioscience Inc. West Roxbury MA USA

**Keywords:** colorectal cancer, irinotecan resistance, ixazomib, proteasome inhibitor, SN‐38, topoisomerase I

## Abstract

Irinotecan, a topoisomerase I (topoI) inhibitor, is widely used for colorectal cancer, but resistance remains a major clinical challenge. We previously showed that camptothecin induces ubiquitin–proteasome pathway (UPP)‐mediated topoI degradation. In this study, we investigated whether inhibition of UPP could prevent topoI degradation and restore camptothecin sensitivity. SN‐38, an active metabolite of irinotecan, induced topoI degradation in irinotecan‐resistant colorectal cancer cell lines, which was suppressed by ixazomib. The combination significantly enhanced cytotoxicity, colony inhibition, and reduced IC_50_ values compared with SN‐38 alone. Mechanistically, ixazomib prevented proteasome‐mediated degradation of ubiquitinated topoI, restoring its stability. *In vivo,* the combination significantly suppressed tumor growth in a DLD‐1 xenograft model compared with SN‐38 alone. These findings indicate that UPP‐dependent topoI degradation is a key mechanism underlying irinotecan resistance in colorectal cancer. Pharmacological inhibition of the proteasome effectively prevents topoI loss and restores irinotecan sensitivity, suggesting that proteasome inhibitors such as ixazomib may serve as promising therapeutic partners for camptothecin‐based chemotherapy.

## Graphical abstract

Ixazomib inhibits proteasome‐mediated degradation of topoisomerase I induced by irinotecan, thereby restoring drug sensitivity and promoting tumor cell death in colorectal cancer.

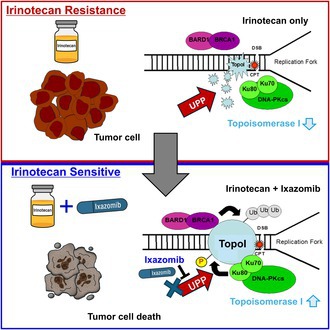

**Abbreviations**
ABC: ATP‐binding cassette
MDR: multidrug resistance
PBS: phosphate‐buffered saline
PI: proteasome inhibitors
SEM: standard error of the mean
sgRNA: A single guide RNA
TCGA: The Cancer Genome Atlas
Topo‐I: topoisomerase I
topoI‐pS10: phosphorylated topoI
UPP: ubiquitin–proteasome pathway

## 1 Introduction

Colorectal cancer is the third most commonly diagnosed malignancy and the second leading cause of cancer‐related mortality worldwide [1]. Approximately 25% of newly diagnosed colorectal cancer patients present with metastatic disease, and among those who undergo curative resection, up to 40% subsequently develop local or distant recurrence [2, 3]. Therefore, chemotherapy remains the cornerstone treatment for advanced or recurrent colorectal cancer.

Camptothecin, a specific inhibitor of topoisomerase I (topoI), is widely used to treat various solid tumors [4]. In advanced or recurrent colorectal cancer, camptothecin and its derivative, irinotecan, are among the standard first‐line agents typically administered as part of the FOLFIRI or FOLFOXIRI regimens, together with 5‐fluorouracil (5‐FU), leucovorin, and oxaliplatin. These regimens are often combined with targeted agents such as anti‐VEGF or anti‐EGFR antibodies, or with immune checkpoint inhibitors, depending on the molecular profile, including RAS, BRAF, MSI, and TMB status [5, 6]. Although these multimodal approaches have significantly extended overall survival to approximately 30 months, the median progression‐free survival of first‐line therapy remains at approximately 10 months, and a subset of patients inevitably develop chemoresistance [7, 8].

Several mechanisms have been proposed to explain camptothecin resistance, including the activation of ATP‐binding cassette (ABC) transporters and multidrug resistance (MDR) genes, mutations in topoI, and enhanced degradation of topoI via the ubiquitin–proteasome pathway (UPP). Large‐scale pharmacogenomic analyses, such as the NCI‐60 cell line panel, have identified limited correlations between topoI expression and drug response. Notably, no association has been observed between camptothecin sensitivity and MDR/ABC transporter gene expression [9, 10]. Although 13 types of topoI point mutations have been reported, they are rarely detected in clinical colorectal cancer specimens, suggesting that topoI mutations are not the dominant mechanism underlying camptothecin resistance [11, 12, 13].

Rubin et al. demonstrated that in leukocytes isolated from patients enrolled in 9‐nitro‐camptothecin clinical trials, the topoI protein level starts to decrease after 24 h of therapy. It was further reduced after 48 h and was almost undetectable by immunoblotting 72 h post‐therapy [14]. This work was followed by several laboratory studies that demonstrated that topoI is degraded in cancer cells and that the rate of degradation varies between different types of cells [15]. Most importantly, Liu *et al*. demonstrated that cells that degrade rapidly are resistant to topoI inhibitors and that the ubiquitin proteasomal pathway is responsible for camptothecin‐induced topoI degradation [16]. However, the underlying molecular determinants were not identified. In our efforts to understand the regulation of topoI, we demonstrated that dysregulated DNA‐PKcs are at the core of camptothecin resistance. In resistant cells, activated DNA‐PKcs maintain a high level of topoI‐S10 phosphorylation, and phosphorylated S10 is rapidly ubiquitinated by BRCA1 and degraded by the proteasome [17].

Therefore, we focused on proteasome inhibitors as potential agents to block UPP‐mediated topoI degradation. Based on these findings, we hypothesized that the pharmacological inhibition of the UPP pathway using ixazomib, an orally bioavailable second‐generation proteasome inhibitor, could prevent camptothecin‐induced topoI degradation, thereby overcoming irinotecan resistance in colorectal cancer.

## 2 Material and methods

### 2.1 Cell culture and drug treatment

Human colorectal cancer cell lines DLD‐1 (RRID: CVCL_0248; JCRB9090), HCT‐15 (RRID: CVCL_0292; ATCC CCL‐225), and HCT‐116 (RRID: CVCL_0291; ATCC CCL‐247) were obtained from the Japanese Cancer Research Resources Bank (JCRB, Tokyo, Japan) and the American Type Culture Collection (ATCC, Manassas, VA, USA), respectively. Cells were cultured in RPMI‐1640 medium (Gibco, Thermo Fisher Scientific, Waltham, MA, USA) supplemented with 10% fetal bovine serum, 2 mm l‐glutamine, 100 U·mL^−1^ penicillin, and 100 μg·mL^−1^ streptomycin. The cells were maintained at 37 °C in a humidified incubator with 5% CO_2_. All cell lines were authenticated by short tandem repeat (STR) profiling and confirmed to be free of mycoplasma contamination using the MycoAlert™ assay (Lonza, Basel, Switzerland). TopoI inhibition was performed using irinotecan (Sigma‐Aldrich, St. Louis, MO, USA), irinotecan (Tocris Bioscience, Bristol, UK), or SN‐38 (Tocris Bioscience) at the indicated concentrations. For proteasome inhibition, cells were treated with ixazomib (MLN9708; kindly provided by Takeda Pharmaceutical Co., Ltd., Osaka, Japan), MLN2238 (AdipoGen Life Sciences, San Diego, CA, USA), bortezomib, or carfilzomib (AdipoGen Life Sciences).

### 2.2 Western blotting analysis

After cell collection, total protein was extracted using RIPA buffer supplemented with protease and phosphatase inhibitors. The lysates were heated at 95 °C for 5 min and subjected to electrophoresis using SuperSep™ Ace 10% gels (Fujifilm, Tokyo, Japan) at 20 mA for 80 min. Proteins were transferred onto PVDF membranes using a Trans‐Blot Turbo Transfer System (Bio‐Rad, Hercules, CA, USA). Primary and secondary antibodies were diluted in iBind™ solution (Invitrogen, Waltham, MA, USA) according to the manufacturer's protocol. The primary antibodies used were mouse anti‐human topoI (clone C‐21, BD Pharmingen™, San Diego, CA, USA; 1:1000; RRID: AB_395843) and rabbit anti‐β‐actin (GeneTex, Irvine, CA, USA; 1:2000; RRID: AB_11174537).

For analysis of NF‐κB–related signaling, the following primary antibodies were additionally used: rabbit monoclonal anti‐NF‐κB p65 (Cell Signaling Technology, Danvers, MA, USA; #8242; RRID: AB_10859369), rabbit monoclonal anti‐phospho‐IκBα (Ser32) (Cell Signaling Technology; #2859; RRID: AB_561111), rabbit monoclonal anti‐phospho‐NF‐κB p65 (Ser536) (Cell Signaling Technology; #3033; RRID: AB_331284), and rabbit monoclonal anti‐IκBα (Cell Signaling Technology; #4814; RRID: AB_10694498).

The secondary antibodies were HRP‐conjugated goat anti‐mouse IgM (μ‐chain specific; RRID: AB_2338535) and HRP‐conjugated goat anti‐rabbit IgG H&L (Abcam, Cambridge, UK; 1:5000; RRID: AB_955439). Protein bands were visualized using a chemiluminescent HRP substrate (Denville Scientific, Holliston, MA, USA) and imaged using an Amersham Imager 600 (GE Healthcare, Chicago, IL, USA).

### 2.3 Colony formation assay

Cells were seeded in six‐well flat‐bottom plates in RPMI‐1640 medium supplemented with 10% fetal bovine serum, 100 U·mL^−1^ penicillin, and 100 μg·mL^−1^ streptomycin. After cell attachment, the indicated drugs were added and the cells were cultured for 10 days, with the medium replaced every 3 days. The colonies were washed with phosphate‐buffered saline (PBS), fixed with 4% paraformaldehyde for 10 min, and stained with Diff‐Quik (Sysmex, Kobe, Japan). Visible colonies containing more than 50 cells were counted using imagej (RRID: SCR_003070).

### 2.4 Integrating EGFP following the hTOP1 gene in HCT‐15 cells and quantifying EGFP intensity using a plate reader

As described by Ando et al. [17], genome‐edited HCT‐15 cells expressing C‐terminally tagged topoI–EGFP were generated by CRISPR–Cas9–mediated homologous recombination. Briefly, ~1 kb of the 5′ and 3′ flanking regions surrounding the hTOP1 stop codon were PCR‐amplified, cloned, and sequence‐verified to exclude cell line–specific polymorphisms. A single guide RNA (sgRNA) was designed to target the hTOP1 stop codon, and a donor plasmid containing an EGFP–P2A–puromycin resistance cassette flanked by homology arms was assembled using Gibson cloning. HCT‐15 cells were co‐transfected with Cas9, sgRNA, and donor plasmids using lipofectamine (Thermo Fisher Scientific). After puromycin selection and cell sorting, the correct integration of the EGFP cassette was confirmed by PCR and Sanger sequencing. Genome‐edited topoI‐EGFP HCT‐15 cells were cultured in black, clear‐bottom 96‐well plates and treated with 2.5 μm SN38 for the indicated time points. After incubation, the medium was replaced with PBS, and EGFP fluorescence intensity was quantified using a plate reader (Tecan).

### 2.5 Immunofluorescence

Cells were grown on sterilized coverslips. After treatment, the coverslips were washed twice with ice‐cold PBS, fixed with 3.7% formaldehyde, and permeabilized with 0.2% Triton X‐100. After blocking with 3% BSA for 60 min, the coverslips were incubated with monoclonal anti‐topoI S10 followed by a secondary antibody, Alexa Fluor™ 568‐conjugated goat anti‐mouse IgG (Molecular Probes; RRID: AB_2534072, Waltham, MA, USA). Cell nuclei were stained with DAPI. Fluorescence imaging was performed using a Leica SP5 fluorescence microscope driven by the LAS software (Leica Microsystems, Wetzlar, Germany).

### 2.6 Cell viability assay

Cell viability was assessed using the Alamar Blue assay to evaluate drug sensitivity quantitatively. HCT‐15, HCT‐116, and DLD‐1 cells were seeded in 96‐well plates at a density of 700–2000 cells per well in 100 μL of complete medium. After cell attachment, the cells were treated with varying concentrations of SN‐38 and ixazomib (IXA), either alone or in combination, and incubated for 72 h at 37 °C in a humidified atmosphere containing 5% CO_2_. Subsequently, 10 μL of Alamar Blue reagent (Invitrogen) was added to each well and incubated for 1 h. Fluorescence intensity was measured using a microplate reader (Tecan, Männedorf, Switzerland) at an excitation wavelength of 560 nm and an emission wavelength of 590 nm. Relative cell viability was calculated using the untreated control as 100%. Data are presented as the mean ± standard error of the mean (SEM) from three independent experiments.

### 2.7 Flow cytometric analysis for sub‐G1 population

Apoptotic cell death was evaluated by analyzing the Sub‐G1 population using a FACS Calibur flow cytometer (Becton Dickinson, Franklin Lakes, NJ, USA). Data were analyzed using flowjo software (RRID: SCR_008520). After drug treatment, cells were harvested, washed twice with ice‐cold PBS, and fixed in 70% ice‐cold ethanol at 4 °C overnight. Fixed cells were washed with PBS and treated with 500 μg·mL^−1^ RNase A (Sigma‐Aldrich, St. Louis, MO, USA) at 37 °C for 30 min. The cells were subsequently stained with 50 μg·mL^−1^ propidium iodide (Sigma‐Aldrich) at 37 °C for 15 min. Data were analyzed with flowjo software (Tree Star, Ashland, OR, USA). The percentage of Sub‐G1 cells was quantified as an indicator of apoptosis.

### 2.8 Mouse xenograft model

Five‐week‐old female BALB/c‐nu/nu mice (Jackson Laboratory Japan, Kanagawa, Japan) were purchased and maintained under specific pathogen‐free conditions at 22 °C with a 12‐h light/dark cycle, with free access to CL‐2 diet (CLEA Japan, Tokyo, Japan) and water. After a one‐week acclimation period, each mouse was subcutaneously inoculated with 1.0 × 10^6^ DLD‐1 cells during the light phase.

Four weeks after inoculation, when tumors reached approximately 5 mm in diameter, mice were randomly assigned into four treatment groups (*n* = 6 per group): a control group receiving vehicle (saline and DMSO) orally three times per week and intraperitoneally once per week for 2 weeks; an ixazomib group (7.0 mg·kg^−1^, orally, three times per week for 2 weeks); an irinotecan group (10 mg·kg^−1^, intraperitoneally, once per week for 2 weeks); and a combination group receiving ixazomib (7.0 mg·kg^−1^, orally) followed 6 h later by irinotecan (10 mg·kg^−1^, intraperitoneally), once weekly for 2 weeks, with additional oral ixazomib dosing (7.0 mg·kg^−1^) twice weekly for 2 weeks. The ixazomib dose was determined based on previously published *in vivo* studies demonstrating antitumor efficacy and tolerability.

Body weight and tumor dimensions were measured three times per week (Days 2, 4, 7, 9, 11, and 14 after treatment initiation), and tumor volume (mm^3^) was calculated as (length × width^2^)/2. For growth curve analysis, tumor progression was expressed as the change in tumor volume from baseline (Δvolume), defined as tumor volume at each time point minus tumor volume at Day 0. Mice showing no tumor engraftment or health deterioration unrelated to treatment were excluded from analysis. Mice were monitored regularly and euthanized at the experimental endpoint (Day 14) or earlier if predefined humane endpoints were met.

Humane endpoints were defined as absolute tumor volume exceeding 2 cm^3^ (2000 mm^3^), tumor length exceeding 2 cm, body weight loss exceeding 20% of pre‐inoculation weight, or evidence of ulceration, necrosis, or infection. All animal experiments were performed in accordance with the Guide for the Care and Use of Laboratory Animals (National Institutes of Health) and approved by the Animal Experiment Committee of Kyushu University (approval no. A25‐286‐1).

### 2.9 Immunohistochemistry

Formalin‐fixed, paraffin‐embedded tumor sections (4 μm thick) obtained from harvested xenograft tumors were subjected to immunohistochemical staining. Antigen retrieval and staining were performed according to the manufacturer's instructions. The primary antibody used was rabbit monoclonal anti–topoisomerase I antibody (EPR5375, Abcam, Cambridge, UK; 1:100; RRID: AB_10865889). Antigen retrieval was performed using the Target Retrieval Solution (pH 9.0; Dako, Glostrup, Denmark) by microwave heating at 98 °C for 15 min. The slides were then incubated overnight at 4 °C with the primary antibody and visualized using the Dako EnVision + System (Dako). Mouse kidney tissue was used as positive and negative controls. The slides were digitized using a NanoZoomer whole‐slide imaging system (Hamamatsu Photonics, Hamamatsu, Japan).

### 2.10 Statistical analysis

All statistical analyses were performed using JMP Pro 17 software (SAS Institute, Cary, NC, USA; RRID: SCR_008567). Data are presented as mean ± SEM. For time‐course experiments involving multiple cell lines and time points, statistical analysis was performed using two‐way analysis of variance followed by *post hoc* multiple comparisons. Differences between two groups were analyzed using two‐tailed unpaired Student's *t*‐tests. A *P*‐value <0.05 was considered statistically significant.

### 2.11 TCGA data analysis

Publicly available colorectal adenocarcinoma datasets from The Cancer Genome Atlas (TCGA; COADREAD) were analyzed using the cBioPortal for Cancer Genomics. Mutation frequencies of cancer‐related genes were assessed, and TOP1 mRNA expression (z‐score) was obtained from RNA sequencing data. Patients were dichotomized into high and low TOP1 expression groups based on the median z‐score (cutoff = 0.96). Progression‐free survival and overall survival were compared between groups using the log‐rank test.

## 3 Results

### 3.1 TopoI degradation correlates with SN‐38 resistance in colorectal cancer cell lines

**Fig. 1:**
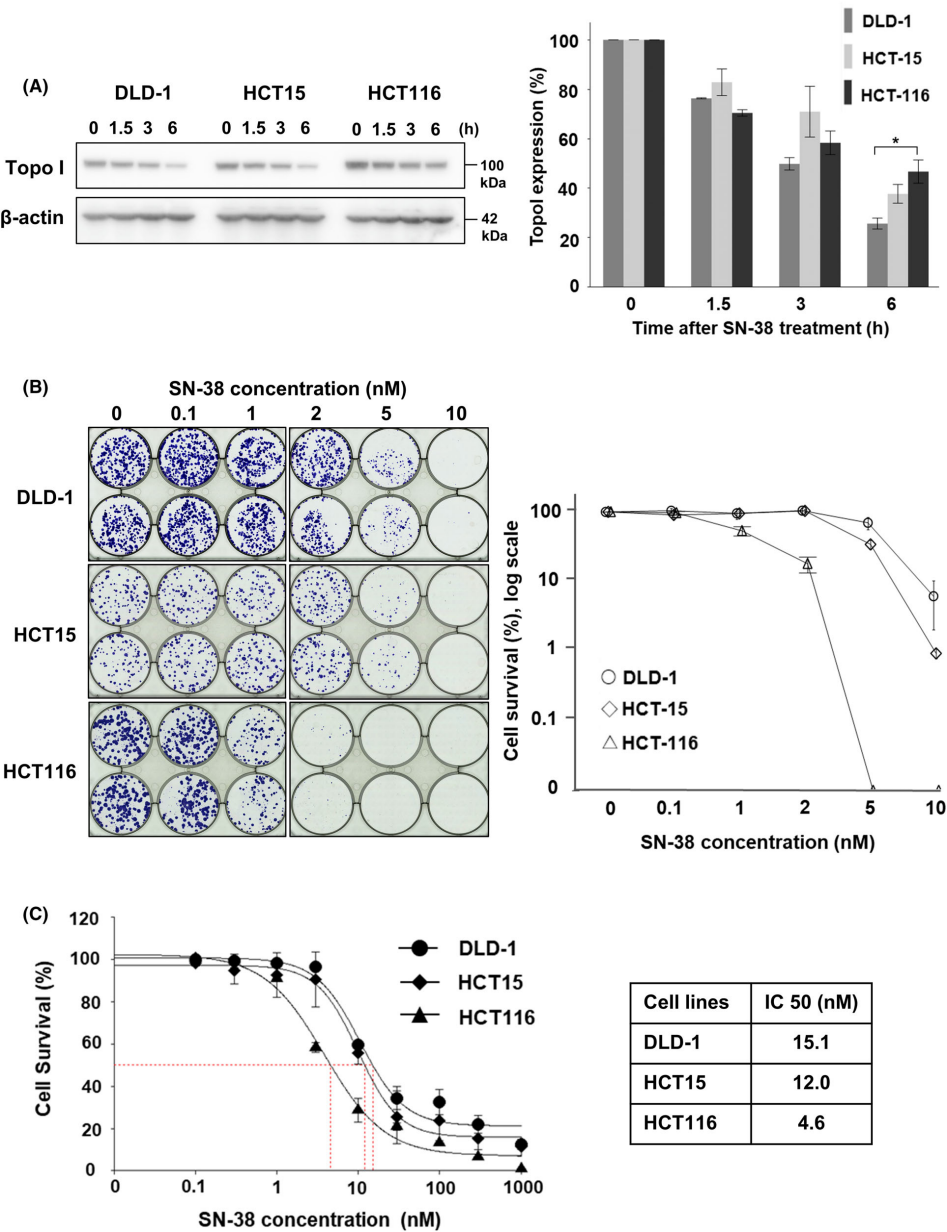
Topoisomerase I degradation correlates with SN‐38 resistance in colon cancer cell lines. (A) Time‐course analysis of topoisomerase‐I (topo‐I) expression in DLD‐1, HCT15, and HCT116 cells treated with 2.5 μm SN‐38. Western blotting was performed at 1.5, 3, and 6 h after treatment. Topo‐I band intensities were quantified by densitometry, normalized to β‐actin, and expressed relative to the 0 h condition (set to 100%) for each cell line. Data are presented as mean ± SEM from three independent experiments. Statistical significance was determined using Student's t‐test. *P = 0.03 indicates a significant difference between DLD‐1 and HCT116 at 6 h. Molecular weight markers (kDa) are indicated. (B) Colony formation assays of DLD‐1, HCT15, and HCT116 cells following exposure to increasing concentrations of SN‐38 (0, 0.1, 1, 2, 5, and 10 nm). Representative images are shown, and quantification of colony survival is presented on the right. Data are presented as mean ± SEM from three independent experiments. No statistical comparison was performed. (C) Dose–response curves of cell survival following SN‐38 treatment. IC_50_ values were calculated for each cell line and are shown in the inset table. The red dashed line indicates 50% cell survival. Data are presented as mean ± SEM from three independent experiments. No statistical comparison was performed.

To investigate the relationship between camptothecin sensitivity and topoI degradation, three colorectal cancer cell lines with distinct drug sensitivities were examined. In camptothecin‐resistant DLD‐1 and HCT15 cells, treatment with 2.5 μm SN‐38 induced a pronounced, and time‐dependent reduction in topoI expression, as detected by western blotting (Fig. 1A). In contrast, camptothecin‐sensitive HCT116 cells showed a less pronounced decrease in topoI levels after drug exposure (*P* < 0.05, Fig. 1A), suggesting that topoI degradation is closely associated with acquired resistance.

The colony formation assay revealed that both DLD‐1 and HCT15 cells maintained substantial clonogenic capacity, even at higher concentrations of SN‐38 (0–10 nm), whereas HCT116 cells exhibited significant growth inhibition under the same conditions (Fig. 1B). Consistent with these observations, the IC_50_ values for SN‐38 were 15.1 nm in DLD‐1, 12.0 nm in HCT15, and 4.6 nm in HCT116, confirming that resistant cell lines display a greater tolerance to SN‐38 (Fig. 1C). Collectively, these findings indicated that UPP‐mediated topoI degradation was closely linked to camptothecin resistance in colorectal cancer cells.

### 3.2 Proteasome inhibition prevents camptothecin‐induced topoI degradation

**Fig. 2:**
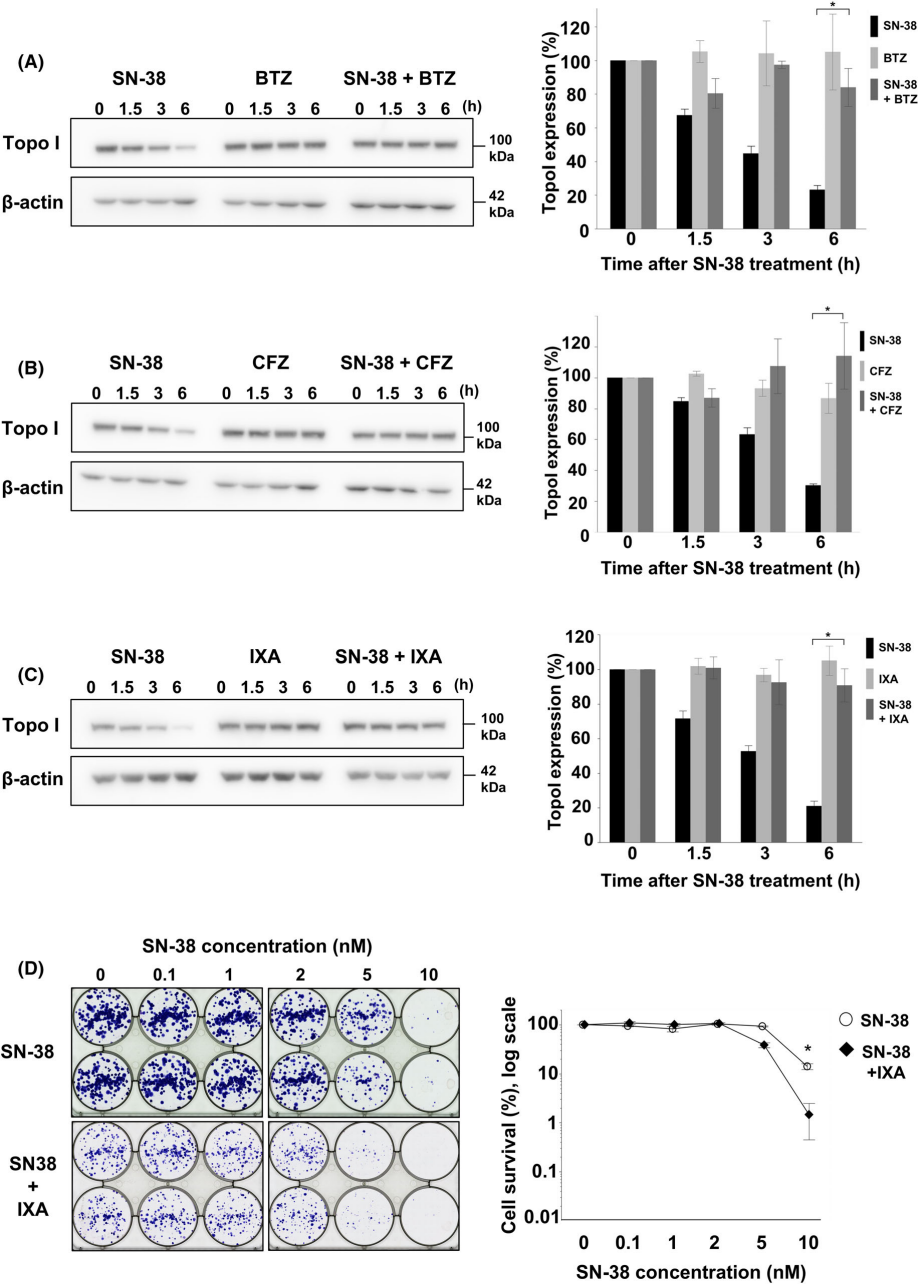
Proteasome inhibitors prevent SN‐38 induced topo‐I degradation in DLD‐1. (A) Western blot analysis of topo‐I expression in DLD‐1 cells treated with SN‐38 (2.5 μm), bortezomib (BTZ, 1 μm), or their combination. Western blotting was performed at 1.5, 3, and 6 h after treatment. Topo‐I band intensities were quantified by densitometry, normalized to β‐actin, and expressed relative to the 0 h condition (set to 100%). Data are presented as mean ± SEM from three independent experiments. Statistical significance was determined using Student's *t*‐test. *P = 0.02 indicates a significant difference between SN‐38 and SN‐38 + BTZ at 6 h. Molecular weight markers (kDa) are indicated. (B) Western blot analysis of topo‐I expression in DLD‐1 cells treated with SN‐38 (2.5 μm), carfilzomib (CFZ, 1 μm), or their combination. Experimental conditions and quantification were performed as described in (A). Data are presented as mean ± SEM from three independent experiments. Statistical significance was determined using Student's *t*‐test. *P = 0.04 indicates a difference between SN‐38 and SN‐38 + CFZ at 6 h. (C) Western blot analysis of topo‐I expression in DLD‐1 cells treated with SN‐38 (2.5 μm), ixazomib (IXA, 1 μm), or their combination. Experimental conditions and quantification were performed as described in (a). Data are presented as mean ± SEM from three independent experiments. Statistical significance was determined using Student's t‐test. *P = 0.03 indicates a significant difference between SN‐38 and SN‐38 + IXA at 6 h. (D) Colony formation assays of DLD‐1 cells treated with increasing concentrations of SN‐38 (0–10 nm) with or without 30 nm IXA. Representative images are shown, and quantification of colony survival is presented on the right. Data are presented as mean ± SEM from three independent experiments. Statistical significance was determined using Student's t‐test. *P = 0.04 indicates a significant difference between SN‐38 and SN‐38 + IXA at 10 nm. Cell survival is expressed as a percentage relative to untreated controls.

Given the association between topoI degradation and camptothecin resistance, we evaluated whether proteasome inhibition could restore topoI stability. In DLD‐1 cells, western blotting showed that treatment with 2.5 μm SN‐38 induced a time‐dependent decrease in topoI expression, whereas co‐treatment with SN‐38 and 1 μm bortezomib prevented topoI degradation (Fig. 2A, *P* < 0.05). Comparable results were obtained using carfilzomib and ixazomib, confirming that topoI degradation occurred via the ubiquitin–proteasome pathway (Fig. 2B,C).

Functionally, the addition of ixazomib significantly enhanced SN‐38 cytotoxicity. In the colony formation assay, SN‐38 alone had limited inhibitory effects on resistant DLD‐1 cells, whereas the combination of SN‐38 and ixazomib markedly reduced colony numbers (Fig. 2D, *P* < 0.05). These findings suggest that proteasome inhibition stabilizes topoI and re‐sensitizes colorectal cancer cells that are resistant to camptothecin derivatives.

### 3.3 Ixazomib restores camptothecin sensitivity in resistant HCT‐15 cells

**Fig. 3:**
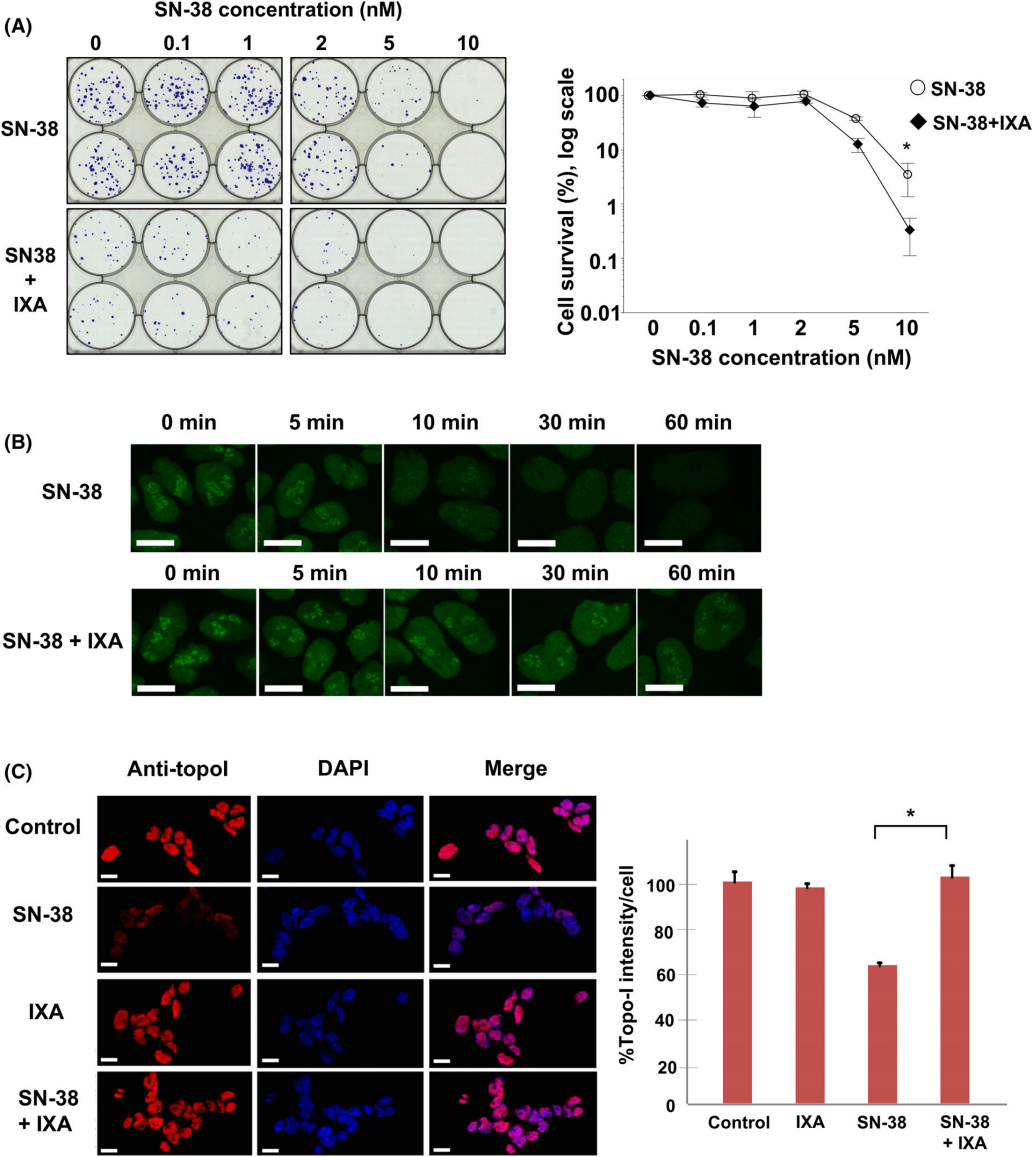
Ixazomib restores SN‐38 sensitivity in resistant HCT‐15 cells. (A) Colony formation assays of HCT‐15 cells treated with increasing concentrations of SN‐38 (0–10 nm) with or without 30 nM IXA. Representative images are shown, and quantification of colony survival is presented on the right. Data are presented as mean ± SEM from three independent experiments. *Cell survival is expressed as a percentage relative to untreated controls. Statistical significance was determined using Student's t‐test. *P* = 0.04 indicates a significant difference between SN‐38 and SN‐38 + IXA. (B) Genetically engineered HCT‐15 cells stably expressing topo‐I–EGFP were treated with 2.5 μm SN‐38 alone or in combination with 1 μm IXA. Time‐lapse fluorescence images were acquired at the indicated time points (0–60 min). Representative images are shown from three independent experiments. Scale bar = 10 μm. (C) Immunofluorescence analysis using anti–topo‐I antibody was performed in HCT‐15 cells treated with DMSO, SN‐38, IXA, or their combination. Nuclei were counterstained with DAPI. Quantification of topo‐I intensity per cell is shown on the right. Data are presented as mean ± SEM from three independent experiments. *Statistical significance was determined using Student's t‐test. *P* = 0.02. Scale bar = 10 μm.

**Fig. 4:**
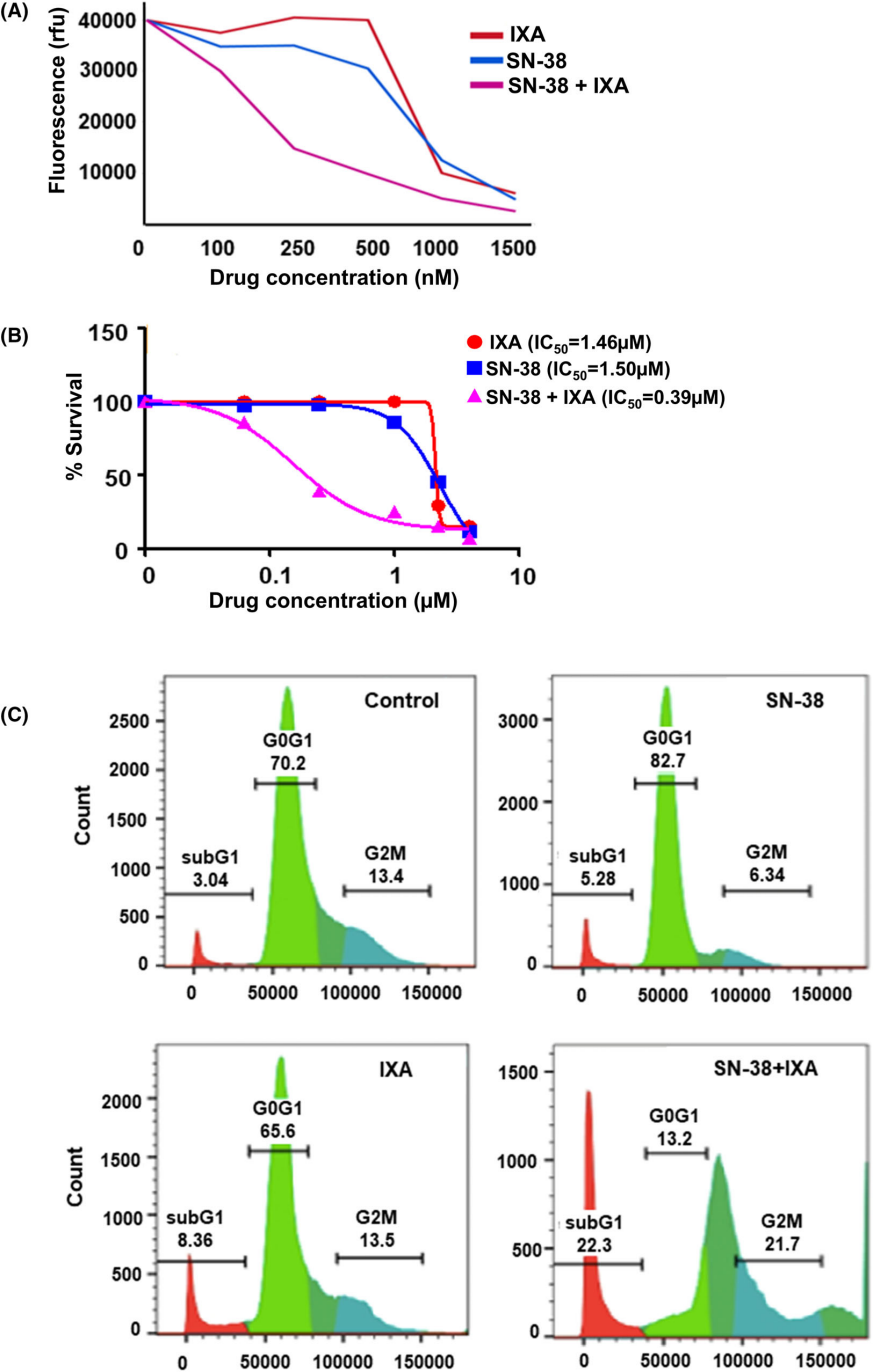
Mechanistic validation of ixazomib‐mediated restoration of SN‐38 sensitivity in HCT‐15 cells. (A) Cell viability assay of HCT‐15 cells treated with increasing concentrations of SN‐38, ixazomib (IXA), or their combination. Cells were seeded in 96‐well plates and treated for 24 h, followed by drug removal. Cell viability was assessed after 72 h using Alamar Blue. Data are presented as mean from three independent experiments. (B) Dose–response curves of HCT‐15 cells treated with SN‐38, IXA, or SN‐38 + IXA. IC_50_ values were calculated by nonlinear regression analysis using GraphPad Prism (version 5.0). Data are presented as mean from three independent experiments. (C) Cell cycle analysis of HCT‐15 cells treated with DMSO (control), SN‐38 (40 nm), IXA (40 nm), or SN‐38 + IXA for 24 h. Cells were harvested after 72 h and analyzed for sub‐G1, G0/G1, and G2/M populations. Representative histograms are shown.

To further clarify the mechanism of the restored sensitivity, we assessed the effect of ixazomib on HCT‐15 cells, which display pronounced camptothecin resistance. In the colony formation assay, SN‐38 alone had limited inhibitory effects on resistant HCT‐15 cells, whereas the combination of SN‐38 and ixazomib markedly reduced colony numbers (Fig. 3A, *P* < 0.05). In genomically edited HCT‐15 cells expressing EGFP‐tagged topoI, SN‐38 alone induced a time‐dependent decrease in topoI (0–60 min), whereas co‐treatment with ixazomib prevented this reduction (Fig. 3B). Immunofluorescence microscopy using an anti‐topoI antibody confirmed these findings: nuclear topoI staining was markedly diminished following SN‐38 exposure but was maintained in the combination group (Fig. 3C, *P* < 0.05). Functionally, co‐treatment with ixazomib significantly reduced cell viability and was associated with an increased apoptotic fraction. The Alamar Blue assays showed a leftward shift in the dose–response curve, indicating increased SN‐38 sensitivity (Fig. 4A,B, *P* < 0.05). Flow cytometric sub‐G1 analysis further demonstrated an increase in the sub‐G1 population in the SN‐38 + ixazomib group compared to the SN‐38 group (Fig. 4C). Taken together, these results demonstrate that ixazomib prevents drug‐induced topoI degradation and restores camptothecin responsiveness in resistant colorectal cancer cells, which is accompanied by reduced cell viability and an increased sub‐G1 fraction.

### 3.4 Combination therapy with irinotecan and ixazomib suppresses tumor growth *in vivo*

**Fig. 5:**
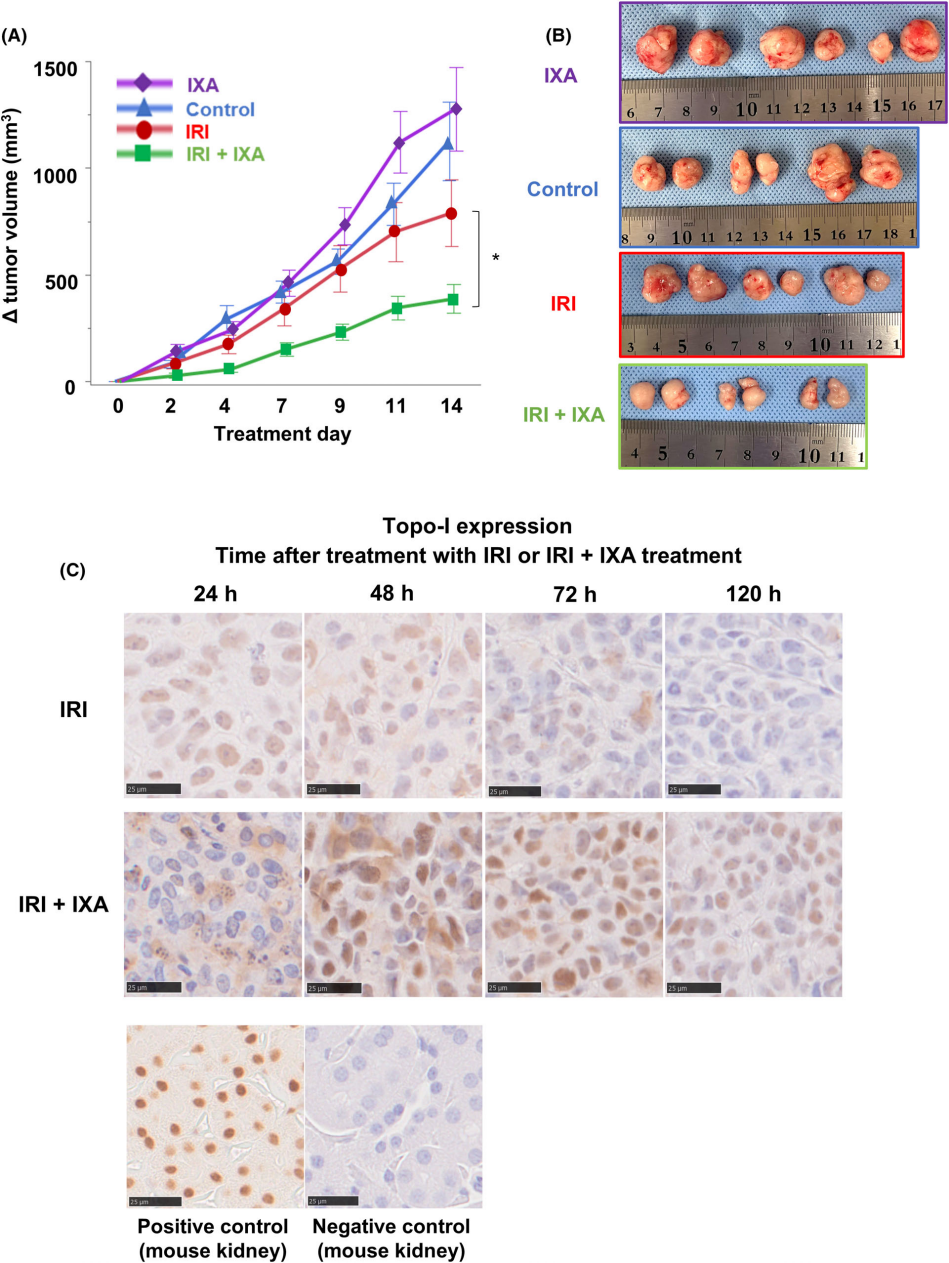
Combination therapy with IRI and IXA suppresses tumor growth in a DLD‐1 xenograft model. (A) BALB/c nude mice were subcutaneously inoculated with 1.0 × 10^6^ DLD‐1 cells. When tumors reached approximately 5 mm in diameter, mice were randomly assigned into four treatment groups (*n* = 6 per group): vehicle control, IXA alone (7.0 mg·kg^−1^, p.o., three times per week for 2 weeks), IRI alone (10 mg·kg^−1^, i.p., once per week for 2 weeks), or the combination (IXA administered 6 h prior to IRI). Tumor volumes were measured on Days 2, 4, 7, 9, 11, and 14 and plotted as Δ tumor volume (mm^3^), defined as tumor volume at each time point minus tumor volume at Day 0. Data are presented as mean ± SEM. *Statistical significance at Day 14 was determined using Student's t‐test comparing the combination group with the IRI‐treated group (*P* = 0.03). Experiments were independently performed twice. (B) Representative tumors harvested on Day 14 after treatment initiation. Numbers below each tumor indicate individual tumor IDs. The maximum absolute tumor volumes at the experimental endpoint were 2488 mm^3^ (control), 2022 mm^3^ (IXA), 1653 mm^3^ (IRI), and 397 mm^3^ (combination). (C) Immunohistochemical staining of topo‐I expression at 24, 48, 72, and 120 h after treatment with IRI alone or in combination with IXA. Images are representative of tumors from each group. Scale bar = 100 μm.

To evaluate the translational relevance of these findings, we investigated the therapeutic efficacy of irinotecan and ixazomib combination therapy *in vivo* using a DLD‐1 xenograft model. Tumor growth was significantly suppressed in the combination group compared with the control, ixazomib‐alone, or irinotecan‐alone groups (Fig. 5A). On Day 14, the mean Δtumor volume (mean ± SEM) was 1093 ± 161 mm^3^ (control), 789 ± 156 mm^3^ (irinotecan), 387 ± 67 mm^3^ (combination: irinotecan and ixazomib), and 1268 ± 193 mm^3^ (ixazomib). The combination group showed a significantly lower Δtumor volume compared with control (*P* = 0.002) and irinotecan (*P* = 0.03). Representative tumors confirmed a marked macroscopic size reduction in the combination arm (Fig. 5B).

Immunohistochemical analysis revealed that topoI expression progressively decreased between 24 and 120 h after irinotecan monotherapy. In contrast, the co‐administration of ixazomib maintained topoI expression during the same period (Fig. 5C). These data indicate that proteasome inhibition enhances the antitumor efficacy of irinotecan *in vivo* by preventing topoI degradation in tumor tissues.

## 4 Discussion

To the best of our knowledge, this is the first report to identify ixazomib as a potential therapeutic partner for camptothecin. In this study, we confirmed that the UPP‐mediated degradation of topoI plays a crucial role in camptothecin resistance in colorectal cancer cells and showed that ixazomib, a proteasome inhibitor, suppresses proteasome‐mediated degradation of ubiquitinated topoI, thereby restoring camptothecin sensitivity.

We demonstrated that camptothecin‐resistant colorectal cancer cell lines (HCT15 and DLD‐1) exhibited more pronounced topoI degradation upon drug exposure, whereas the sensitive HCT116 line showed only partial degradation and retained its drug responsiveness. These findings are consistent with and extend previous reports indicating that UPP‐mediated degradation of topoI is the key mechanism underlying camptothecin resistance [17, 18, 19]. This mechanism has been elucidated as follows: (i) irinotecan inhibits the DNA release step catalyzed by topoI, leading to the generation of DNA double‐strand breaks; (ii) these breaks activate the DNA‐PK complex, which phosphorylates topoI at S10; (iii) BRCA1 binds to phosphorylated topoI and mediates its ubiquitination; and (iv) topoI is subsequently degraded through the UPP [17]. Our results provide functional validation of this pathway in colorectal cancer models, linking the kinetics of topoI degradation directly to drug sensitivity. Taken together, these findings support a model in which SN‐38–induced, proteasome‐mediated topoisomerase I degradation functionally contributes to camptothecin resistance, rather than reflecting a purely intrinsic property of resistant cells.

Consistent with these mechanistic findings, clinical studies have demonstrated that high expression of phosphorylated topoI (topoI‐pS10) in tumor specimens correlates with poor response to camptothecin‐based chemotherapy and that topoI‐pS10 may serve as a predictive biomarker of camptothecin efficacy [20, 21].

To further contextualize these findings in human colorectal cancer, we analyzed publicly available TCGA datasets. TCGA analyses revealed that TOP1 mutations are rare and that TOP1 mRNA expression levels alone do not significantly correlate with clinical outcomes, including progression‐free or overall survival (Fig. S1a–c). These results suggest that genomic or transcriptomic alterations in TOP1 are insufficient to explain clinical irinotecan resistance, highlighting the potential importance of post‐translational regulation such as UPP‐mediated topoI degradation.

In this study, pharmacological blockade of UPP using the second‐generation proteasome inhibitor ixazomib effectively prevented camptothecin‐induced topoI degradation and overcame resistance both *in vitro* and *in vivo*. Proteasome inhibitors (PI), including the first‐generation agent bortezomib and the second‐generation compounds carfilzomib and ixazomib, have been widely used in the treatment of multiple myeloma [22, 23, 24]. Bortezomib has been approved for the treatment of multiple myeloma and mantle cell lymphoma; however, its clinical utility is limited by its significant toxicity and acquired resistance. To overcome these limitations, next generation inhibitors such as carfilzomib and ixazomib were developed [22, 25]. Ixazomib is the first orally bioavailable PI with a favorable safety profile and has been shown to improve progression‐free survival in patients with multiple myeloma [26]. Previous studies demonstrated that bortezomib and carfilzomib enhance the antitumor effects of camptothecin [27, 28]. In this study, we reconfirmed that bortezomib and carfilzomib also suppressed SN‐38–induced topoI degradation when combined with camptothecin, and this effect was consistently observed in both DLD‐1 and HCT‐15 colorectal cancer cells (Fig. 2; Fig. S2a–c), suggesting that these proteasome inhibitors may enhance camptothecin sensitivity by inhibiting topoI degradation.

In addition to the effects on topoisomerase I stability, proteasome inhibition has been reported to modulate NF‐κB signaling in the context of SN‐38 treatment. Consistent with previous studies showing that carfilzomib attenuates SN‐38–induced NF‐κB activation [27], we observed a similar reduction in NF‐κB activation signals upon concomitant treatment with ixazomib and SN‐38. In particular, phosphorylation of NF‐κB p65 was reduced under combination treatment, indicating suppression of NF‐κB activation signals in this setting (Fig. S3a,b). Recent studies have further explored the antitumor activity of ixazomib in esophageal and hepatocellular carcinoma models, underscoring its broad therapeutic potential across solid tumors [24, 29, 30, 31, 32].

Our findings clearly demonstrated that ixazomib co‐treatment stabilizes topoI protein levels and enhances camptothecin cytotoxicity in resistant colorectal cancer cells. In camptothecin‐resistant HCT15 cells, ixazomib restored SN‐38 sensitivity, as confirmed by the colony formation assay, Alamar Blue viability assay, and sub‐G1 apoptotic analysis. Immunofluorescence and live‐cell imaging using Topo I‐EGFP confirmed that ixazomib preserved topoI expression and prevented its degradation following SN‐38 exposure. Furthermore, *in vivo* xenograft experiments using camptothecin‐resistant DLD‐1 cells revealed that the combination of camptothecin and ixazomib significantly inhibited tumor growth, whereas immunohistochemistry confirmed the preservation of topoI expression within tumor tissues. These results are consistent with previous reports combining bortezomib or carfilzomib with camptothecin and suggest that the combination of ixazomib with camptothecin may overcome camptothecin resistance [27, 28]. A phase I clinical trial of ixazomib in combination with the endothelial growth factor inhibitor pazopanib in patients with TP53‐mutant solid tumors, including metastatic colorectal cancer, demonstrated favorable progression‐free survival and overall survival outcomes [33]. These findings support the rationale for the clinical evaluation of camptothecin + ixazomib as a promising therapeutic strategy for overcoming irinotecan resistance in colorectal cancer [34].

This study has several limitations. First, our analyses were primarily conducted in preclinical models, which may not fully recapitulate the tumor microenvironment and interpatient heterogeneity influencing irinotecan response. Second, although the suppression of SN‐38–induced topoisomerase I degradation by proteasome inhibitors was confirmed in two irinotecan‐resistant colorectal cancer cell lines, downstream phenotypic effects were not comprehensively evaluated across multiple models, and apoptosis‐related outcomes were mainly assessed by sub‐G1 analysis. Further studies using patient‐derived organoids and clinical specimens will be important to validate the clinical relevance of these findings, as well as to define the immunomodulatory effects and safety profile of the ixazomib–irinotecan combination. Nevertheless, our findings highlight proteasome inhibition as a promising approach for reversing irinotecan resistance and improving treatment outcomes in patients with colorectal cancer.

## 5 Conclusion

This study functionally confirmed UPP‐dependent topoI degradation as a pivotal mechanism of camptothecin resistance and provided robust preclinical evidence that ixazomib co‐treatment can overcome this resistance.

## Conflict of interest

EO report honoraria from Takeda Pharmaceutical, Ono Pharmaceutical, MSD K.K. Bristol Meyers Squibb and Eli Lilly; and research funding from Guardant Health.

## Author contributions

YE conceived the study, performed the experiments, analyzed and interpreted the data, and wrote the manuscript. KA and ABh conceived and designed the study and supervised the project. HH and EU provided technical support. SP and AT contributed to data interpretation. ABe contributed to the study design. KM provided critical suggestions for the study. EO supervised the study and manuscript preparation. TY contributed to supervised manuscript preparation. All authors reviewed and approved the final manuscript.

## Supporting information


**Fig. S1.** TCGA analysis of TOP1 alterations and mRNA expression in colorectal adenocarcinoma.
**Fig. S2.** Proteasome inhibitors prevent SN‐38 induced topo‐I degradation in HCT‐15.
**Fig. S3.** Time‐dependent changes in NF‐κB‐related signaling molecules after SN‐38 and/or ixazomib treatment.

## Data Availability

Data supporting the findings of this study are available from the corresponding author on reasonable request.
