# Supplementary material for: Proteasome inhibitor, ixazomib prevents topoisomerase‐I degradation and reverses irinotecan resistance in colorectal cancer
Source: Mol Oncol. 2026 Apr 15;20(8):2114–28. doi: 10.1002/1878-0261.70256 (PMC13398833; doi:10.1002/1878-0261.70256)

Supplementary Figure S1

(a)

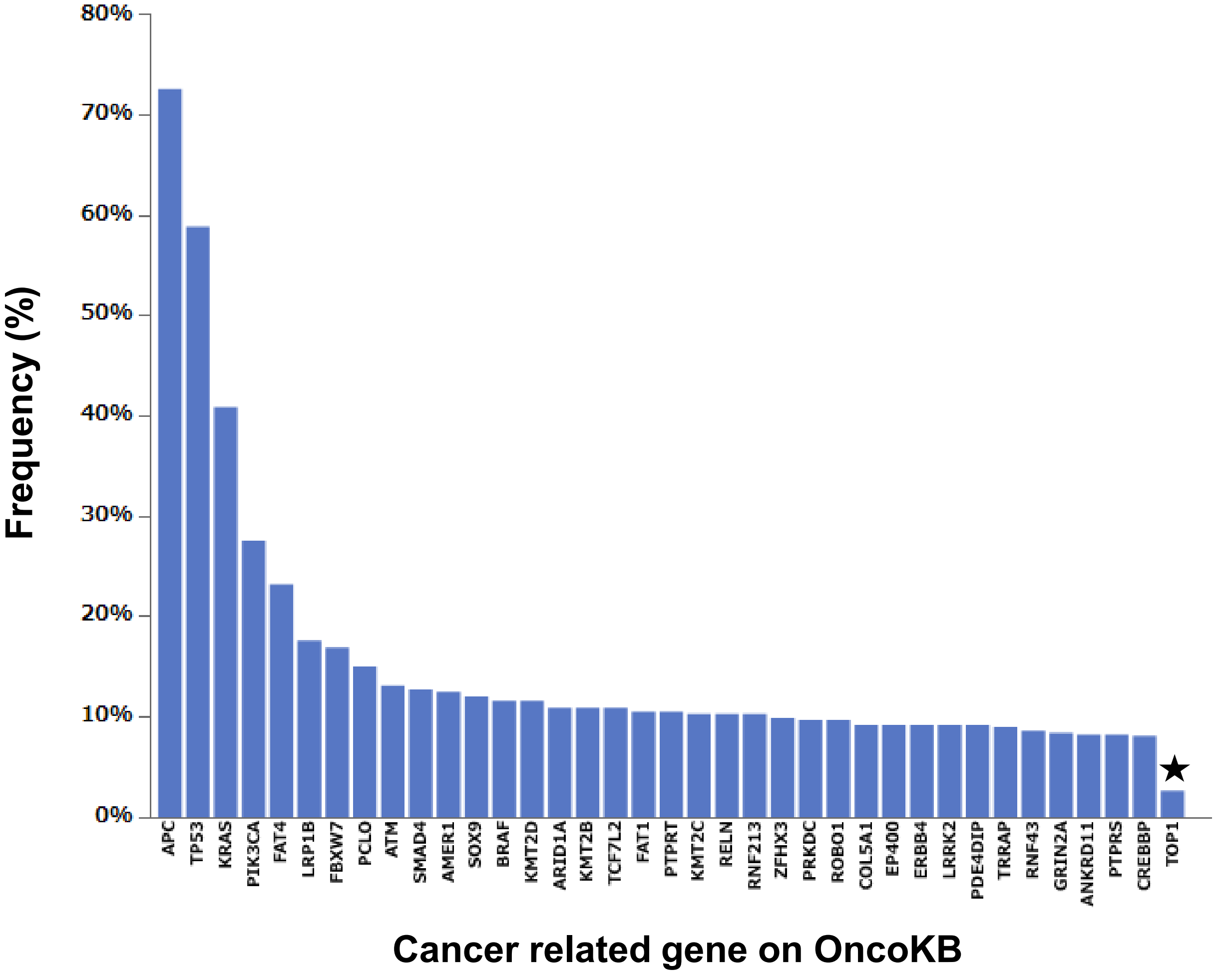

(b)

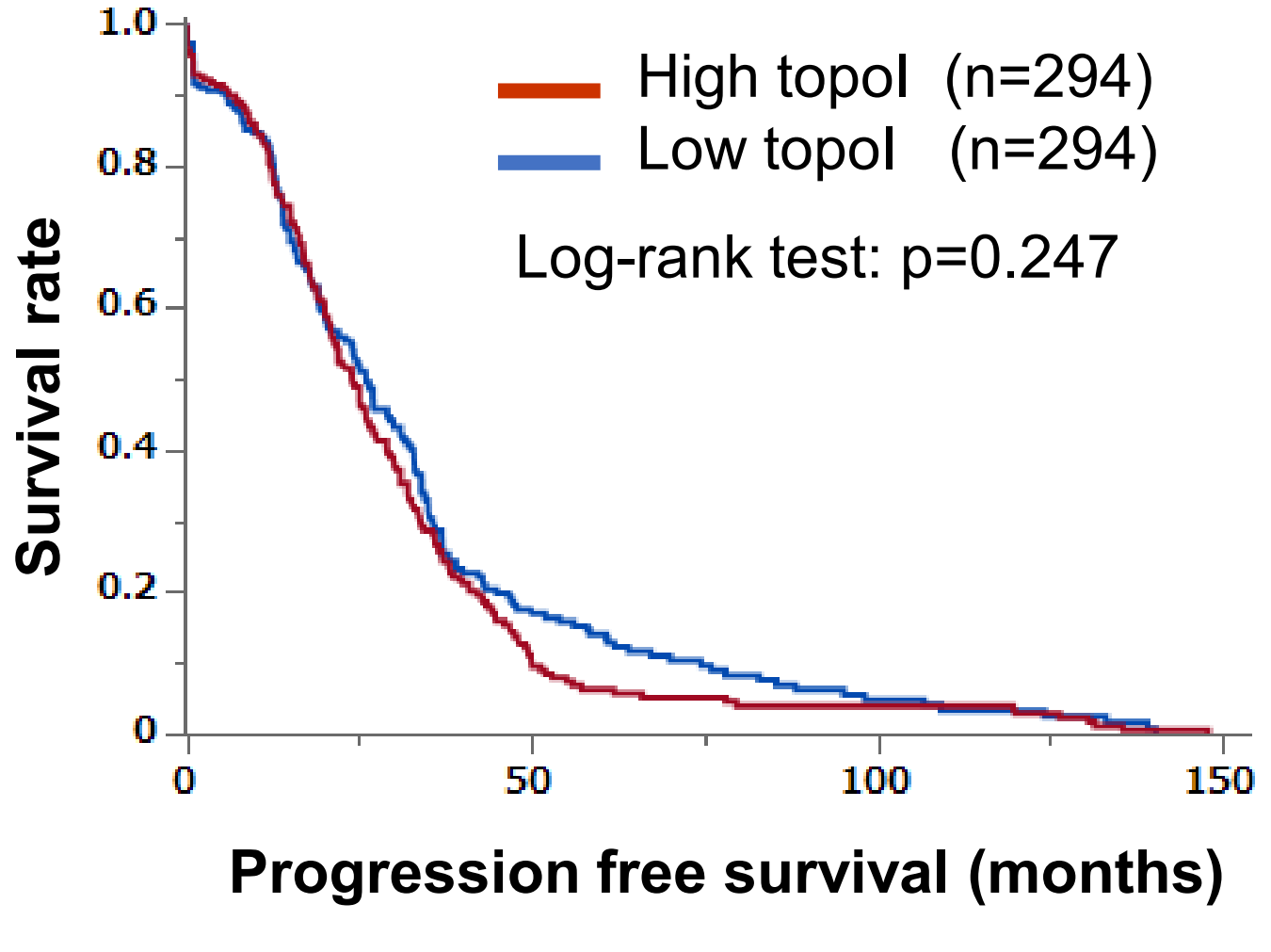

(c)

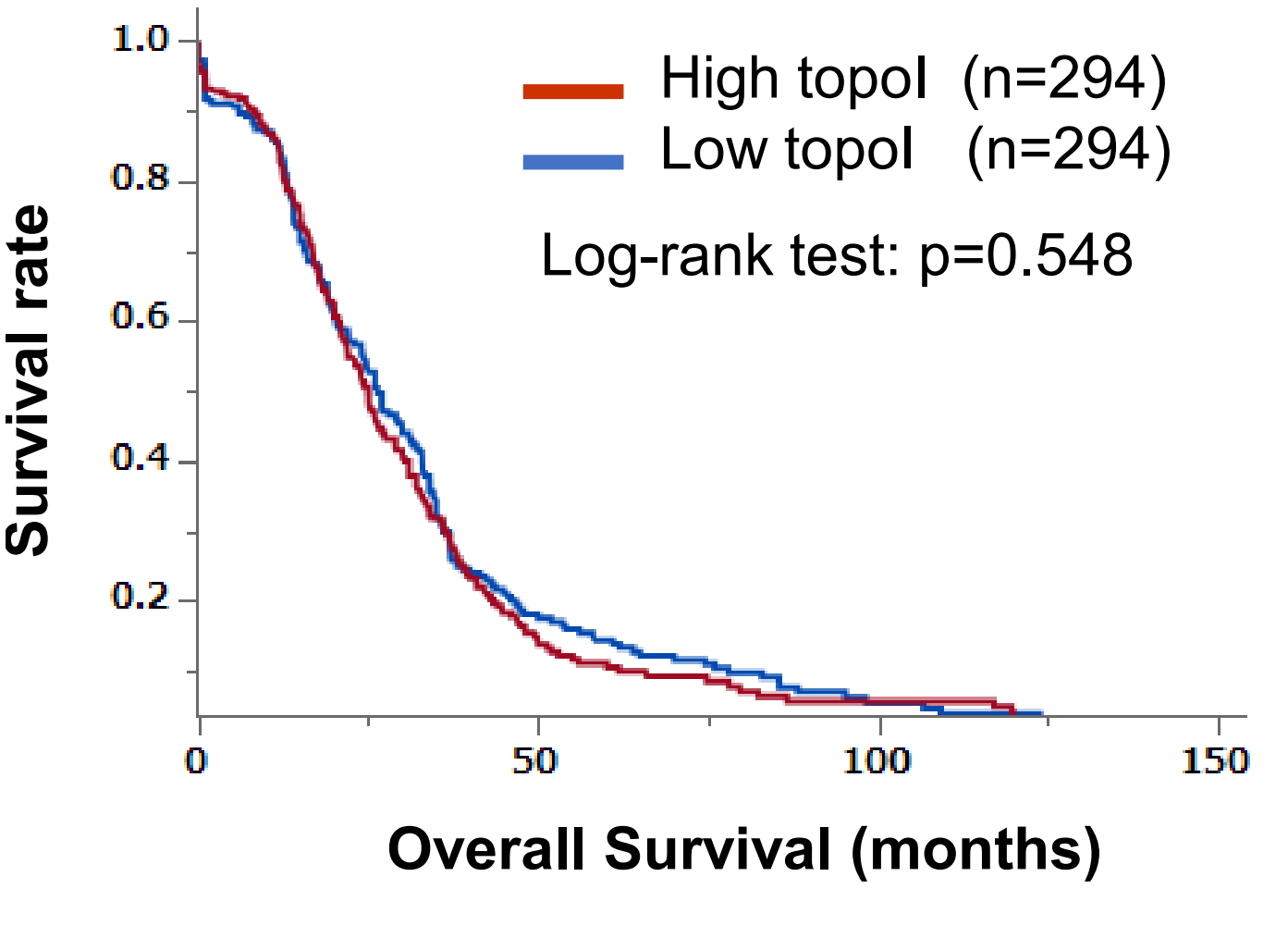

Supplementary Figure S2

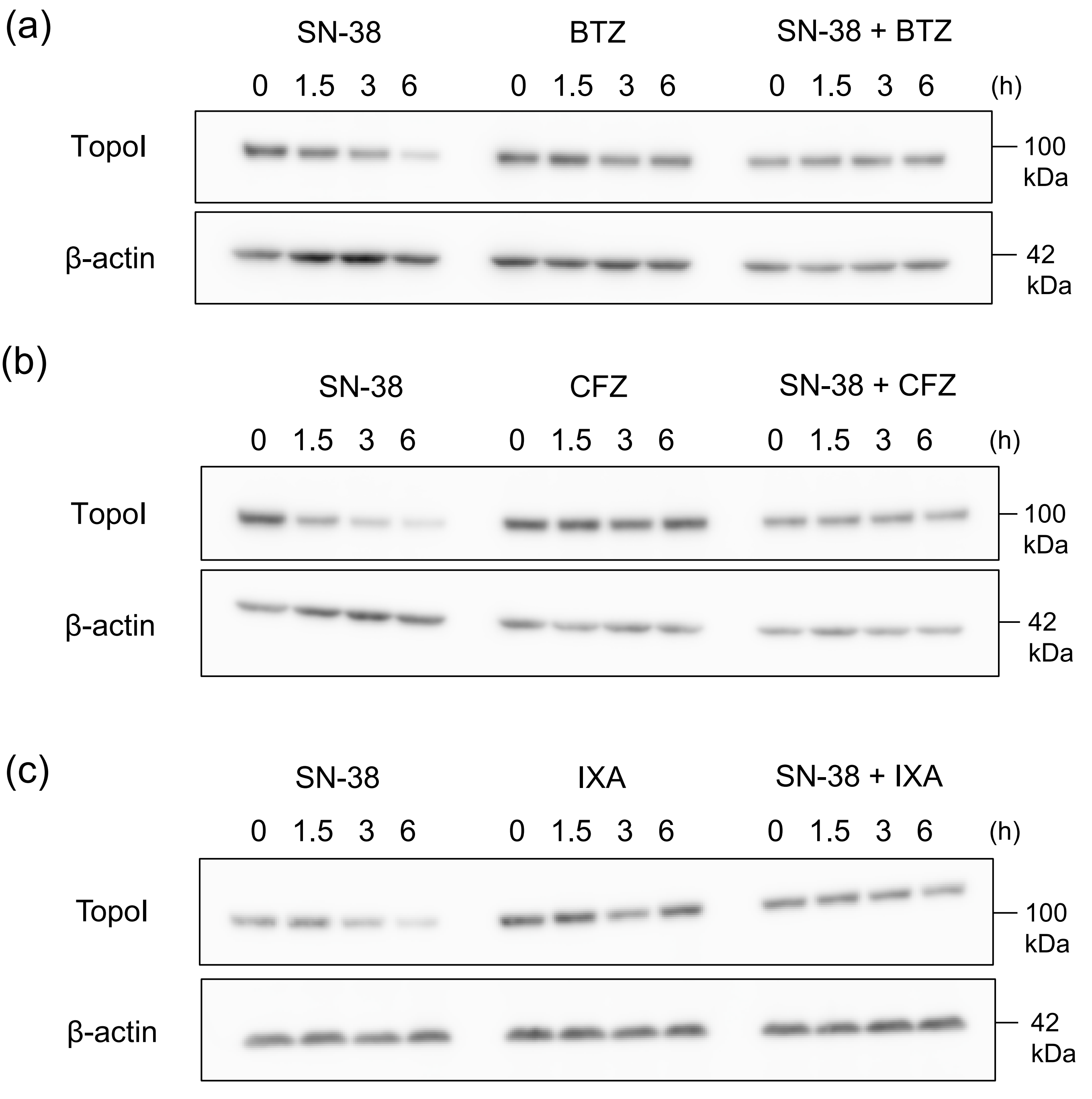

Supplementary Figure S3

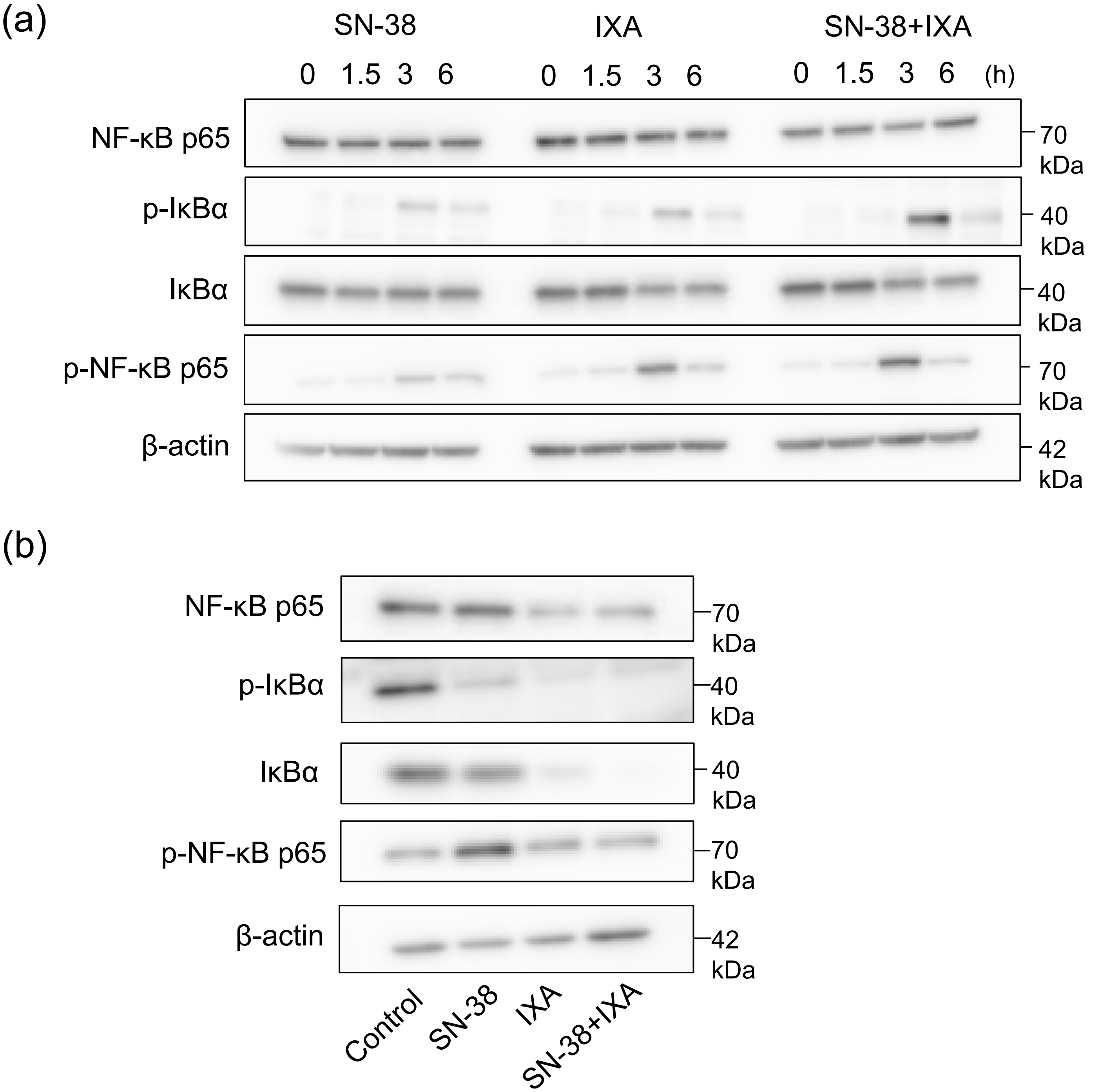

Supplement: Supplementary file 1 [file MOL2-20-2114-s001.pdf]
