# Supplementary figures and images for: Proteasome inhibitor, ixazomib prevents topoisomerase‐I degradation and reverses irinotecan resistance in colorectal cancer
Source: Mol Oncol. 2026 Apr 15:10.1002/1878-0261.70256. Online ahead of print. doi: 10.1002/1878-0261.70256 (PMC13398833; doi:10.1002/1878-0261.70256)

# Supplementary Figure S1

(a)

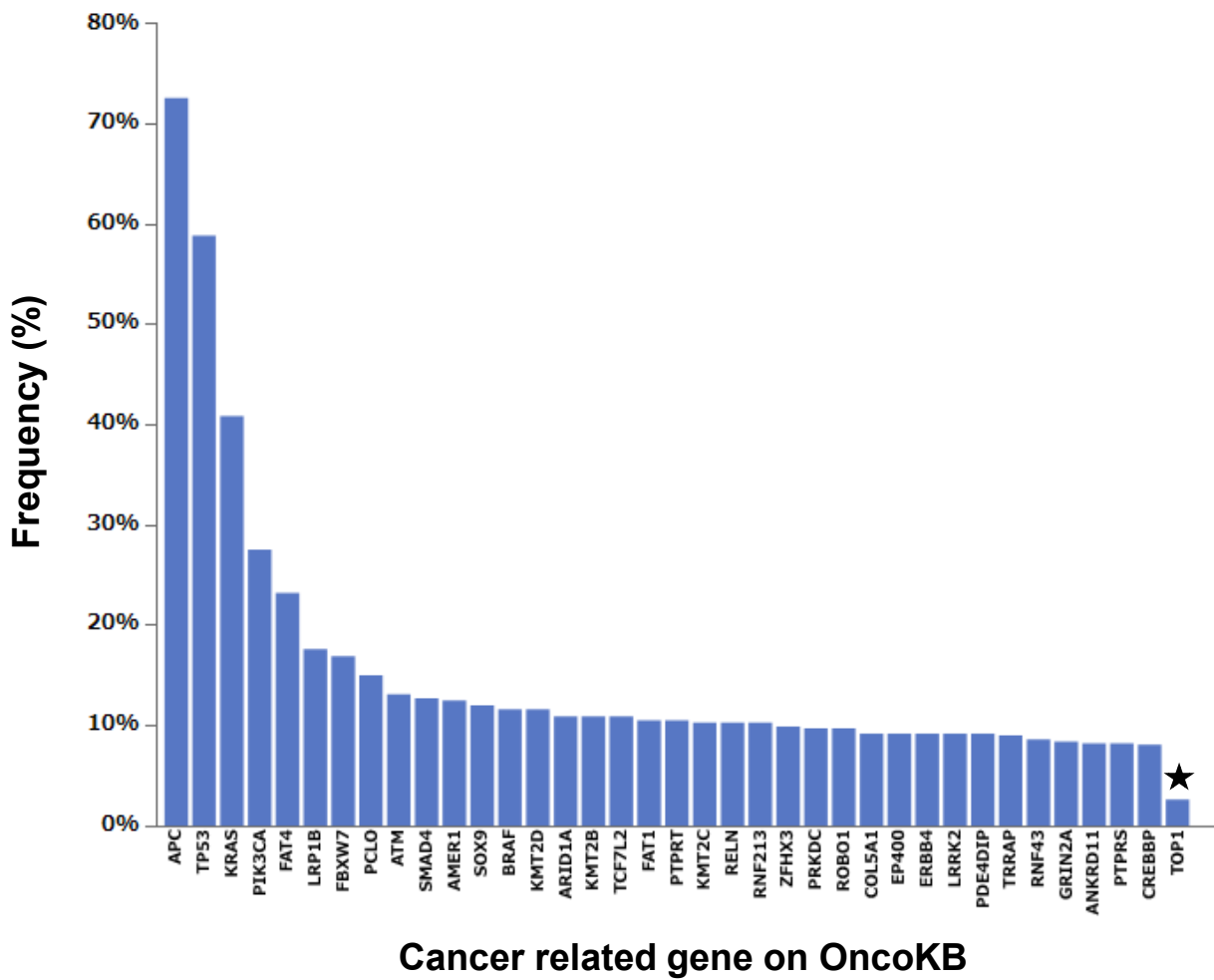

(b)

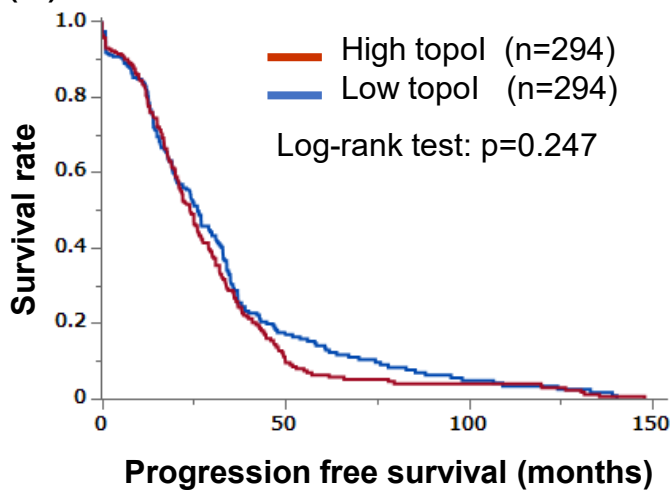

(c)

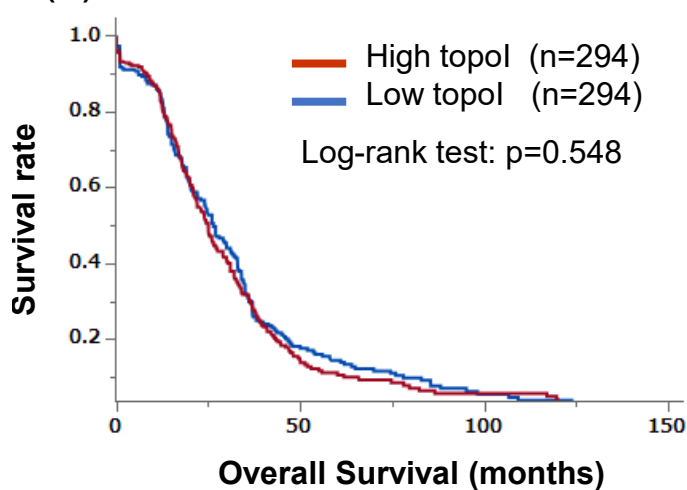

Supplementary Figure S2

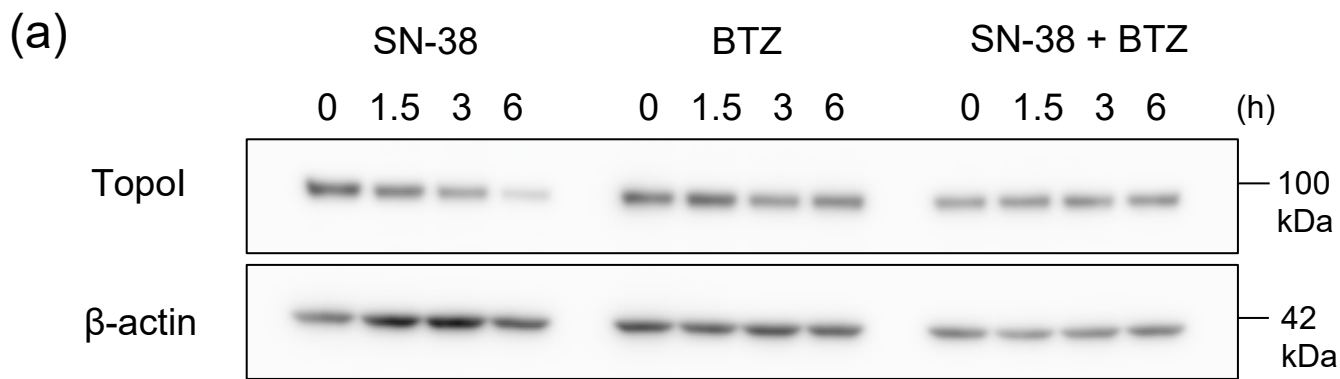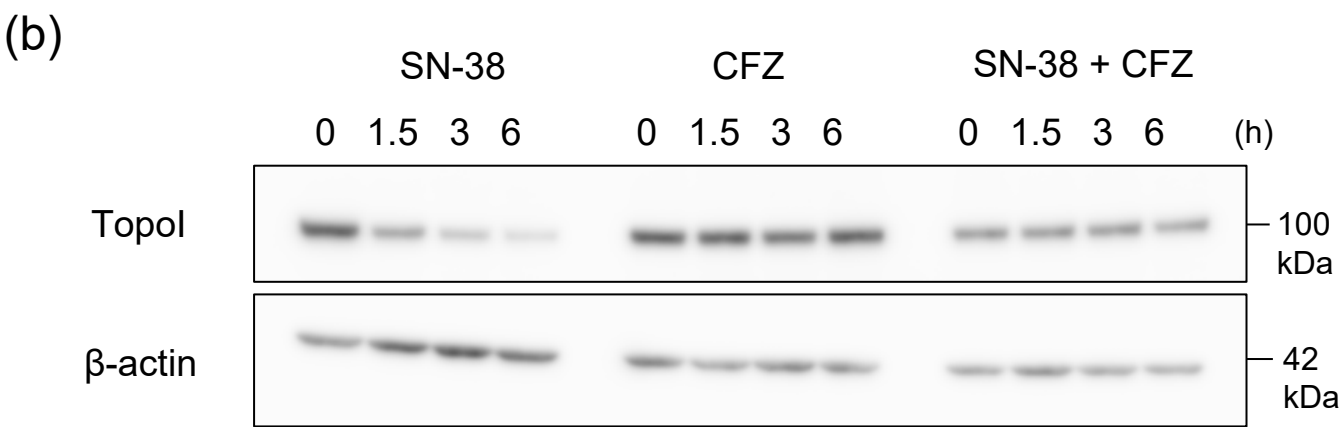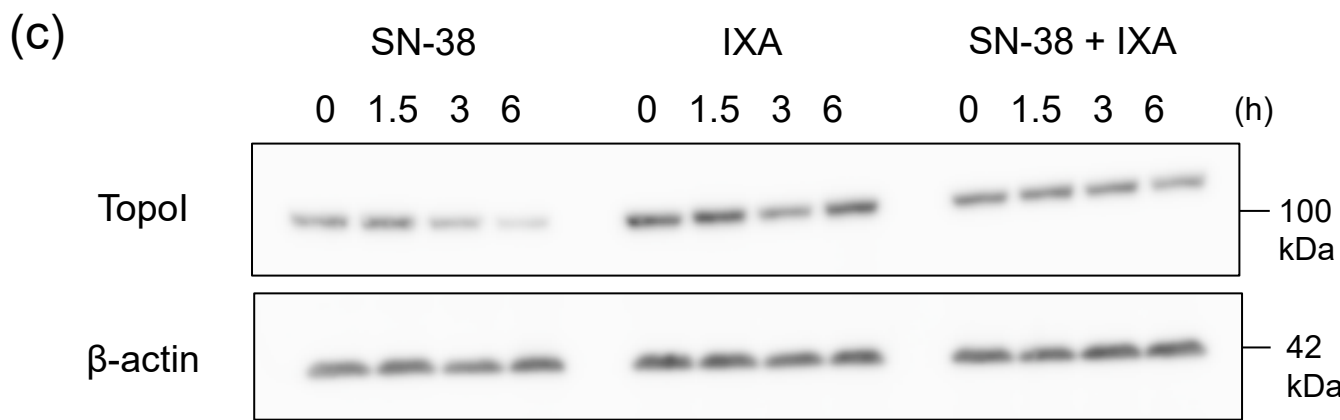

Supplementary Figure S3

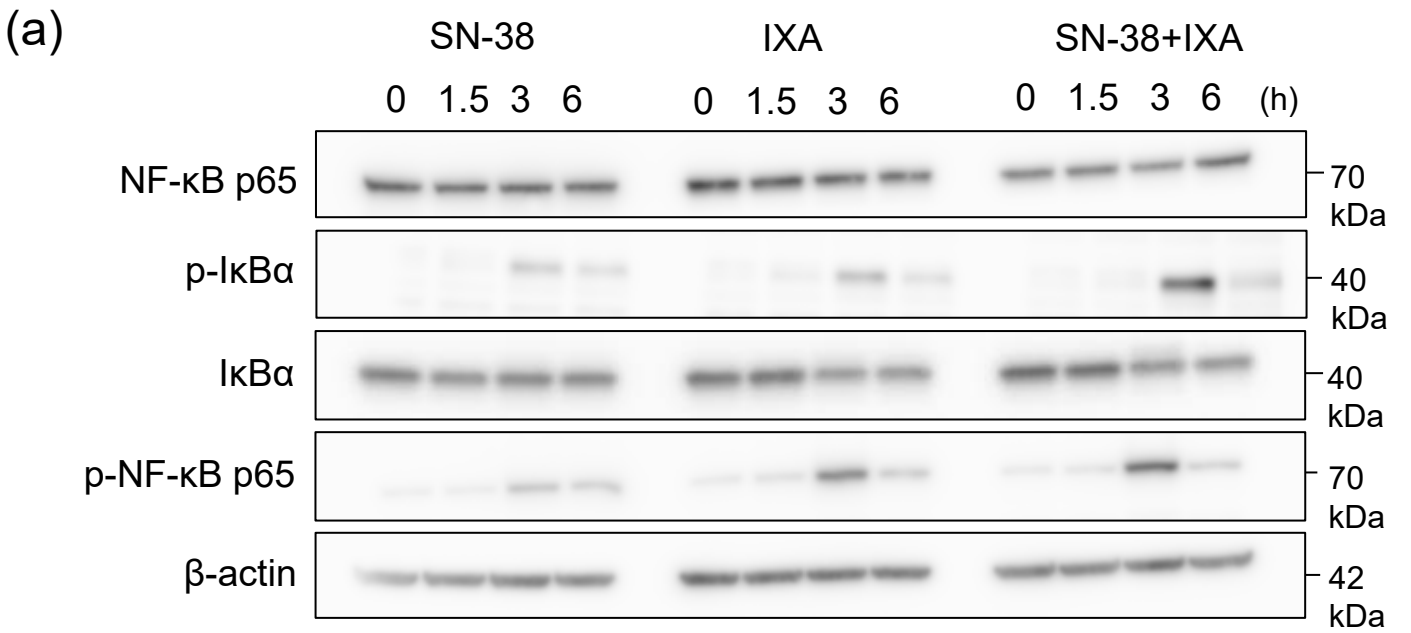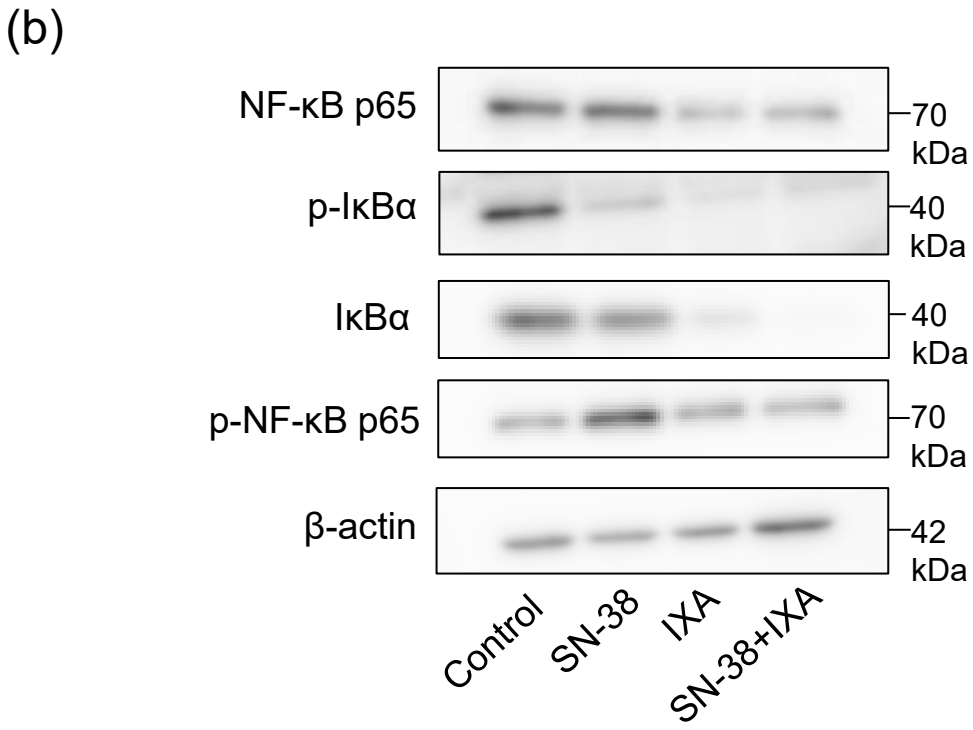

Supplement: Supplementary file 1 — Fig. S1. TCGA analysis of TOP1 alterations and mRNA expression in colorectal adenocarcinoma. Fig. S2. Proteasome inhibitors prevent SN‐38 induced topo‐I degradation in HCT‐15. Fig. S3. Time‐dependent changes in NF‐κB‐related signaling molecules after SN‐38 and/or ixazomib treatment. [file MOL2-9999-0-s001.pdf]
